# Propyl 4-hydroxy­benzoate

**DOI:** 10.1107/S1600536810000139

**Published:** 2010-01-30

**Authors:** Yiwen Zhou, Guzalnur Matsadiq, Yanling Wu, Jing Xiao, Jing Cheng

**Affiliations:** a Key Laboratory of Pesticide & Chemical Biology, Ministry of Education, College of Chemistry, Central China Normal University, Wuhan 430079, People’s Republic of China

## Abstract

There are two mol­ecules in the asymmetric unit of the title compound, C_10_H_12_O_3_. In the crystal, mol­ecules are linked by O—H⋯O hydrogen bonds into chains running along [010]. Adjacent chains are joined together by weak π–π inter­actions between benzene rings [centroid–centroid distance = 4.040 (2) Å].

## Related literature

For the structure of another *p*-hydroxybenzoate, see: Mandal & Kadirvelraj (1996 ▶).
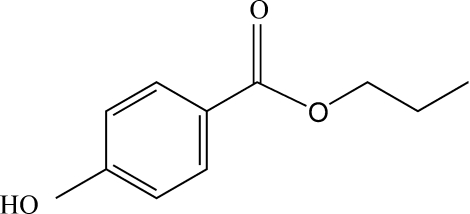


## Experimental

### 

#### Crystal data

C_10_H_12_O_3_
*M* *_r_* = 180.20
Monoclinic, 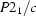

*a* = 12.0634 (12) Å
*b* = 13.8419 (14) Å
*c* = 11.7982 (11) Å
β = 108.625 (2)°
*V* = 1866.9 (3) Å^3^
*Z* = 8
Mo *K*α radiation
μ = 0.09 mm^−1^
*T* = 298 K
0.30 × 0.20 × 0.20 mm

#### Data collection

Bruker SMART APEX CCD area-detector diffractometer
10603 measured reflections
3271 independent reflections
2960 reflections with *I* > 2σ(*I*)
*R*_int_ = 0.027

#### Refinement

*R*[*F* ^2^ > 2σ(*F* ^2^)] = 0.083
*wR*(*F* ^2^) = 0.189
*S* = 1.27
3271 reflections
237 parameters
H-atom parameters constrained
Δρ_max_ = 0.24 e Å^−3^
Δρ_min_ = −0.30 e Å^−3^

### 

Data collection: *SMART* (Bruker, 2001 ▶); cell refinement: *SAINT-Plus* (Bruker, 2001 ▶); data reduction: *SAINT-Plus*; program(s) used to solve structure: *SHELXS97* (Sheldrick, 2008 ▶); program(s) used to refine structure: *SHELXL97* (Sheldrick, 2008 ▶); molecular graphics: *PLATON* (Spek, 2009 ▶); software used to prepare material for publication: *PLATON*.

## Supplementary Material

Crystal structure: contains datablocks I, global. DOI: 10.1107/S1600536810000139/fk2009sup1.cif

Structure factors: contains datablocks I. DOI: 10.1107/S1600536810000139/fk2009Isup2.hkl

Additional supplementary materials:  crystallographic information; 3D view; checkCIF report

## Figures and Tables

**Table 1:** Hydrogen-bond geometry (Å, °)

*D*—H⋯*A*	*D*—H	H⋯*A*	*D*⋯*A*	*D*—H⋯*A*
O4—H4⋯O5^i^	0.82	1.93	2.730 (3)	167
O1—H1⋯O2^ii^	0.82	1.91	2.720 (3)	171

Symmetry codes: (i) 
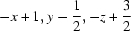
; (ii) 
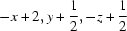
.

**Fig. 1.**
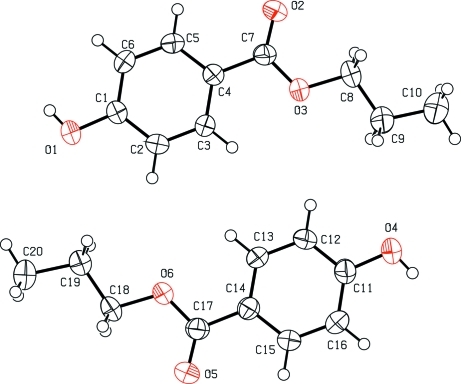
Molecular structure of (I), showing the atom-labeling scheme. Displacement ellipsoids are drawn at the 50% probability level.

**Fig. 2.**
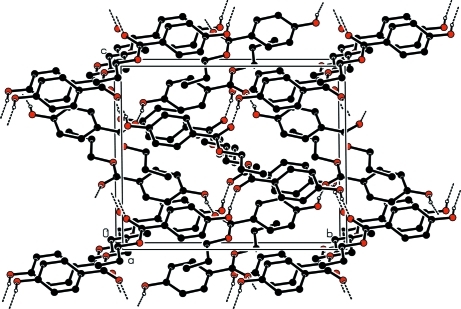
The packing of (I), with O-H···O hydrogen bonds shown as dashed lines.

## supplementary crystallographic information

### Comment

The propyl 4-hydroxybenzoate is a kind of *p*-hydroxybenzoates, which are also known as Nipagin esters. Nipagin ester is a preservative of large consumption in the world. Due to the high antibacterial activity and low toxicity of Nipagin ester, it becomes an inevitable trend that Nipagin ester replaces the traditional preservative. Here, we report the crystal structure of propyl 4-hydroxybenzoate.

There are two molecules in the asymmetric unit (Fig. 1). All bond lengths and bond angles lie in expected ranges.

As shown in Fig.2, molecules are linked by O—H···O hydrogen bonds into one-dimensional chains running along the [010] direction. Adjacent chains are further linked together by weak π–π interactions between two phenyl rings (centroid-to-centroid distance is 4.040 Å).

### Experimental

^1^H-NMR (C_3_D_6_O,600MHZ):δ7.916(d,2H,aromatic),δ6.933(d,2H,aromatic), δ4.208(t,2H,-COOCH_2_-),δ2.096(s,1H,-OH),δ1.752(q,2H,-CH_2_-), δ1.004(t,3H,-CH_3_);

Crystals appropriate for data collection were obtained by slow evaporation of an ethanol solution at room temperature.

### Refinement

All the H atoms attached to carbon atoms were located from geometrical considerations with C—H= 0.93Å (aromatic), 0.97Å (methylene) and 0.96Å (methyl), and *U*_eq_(H)=1.2*U*_eq_(aromatic, methylene C) and 1.5*U*_eq_(methyl C). Hydrogen atoms H1 and H4 were found from difference maps and then placed at their ideal positions with O—H=0.82Å and *U*_iso_(H)=1.5*U*_eq_(O).

### Crystal data

**Table:**

C_10_H_12_O_3_	*F*(000) = 768
*M**_r_* = 180.20	*D*_x_ = 1.282 Mg m^−^^3^
Monoclinic, *P*2_1_/*c*	Mo *K*α radiation, λ = 0.71073 Å
Hall symbol: -P 2ybc	Cell parameters from 4791 reflections
*a* = 12.0634 (12) Å	θ = 2.3–28.3°
*b* = 13.8419 (14) Å	µ = 0.09 mm^−^^1^
*c* = 11.7982 (11) Å	*T* = 298 K
β = 108.625 (2)°	Block, colourless
*V* = 1866.9 (3) Å^3^	0.30 × 0.20 × 0.20 mm
*Z* = 8	

### Data collection

**Table:**

Bruker SMART APEX CCD area-detector diffractometer	2960 reflections with *I* > 2σ(*I*)
Radiation source: fine focus sealed Siemens Mo tube	*R*_int_ = 0.027
graphite	θ_max_ = 25.0°, θ_min_ = 2.3°
0.3° wide ω exposures scans	*h* = −14→12
10603 measured reflections	*k* = −16→16
3271 independent reflections	*l* = −11→14

### Refinement

**Table:**

Refinement on *F*^2^	Primary atom site location: structure-invariant direct methods
Least-squares matrix: full	Secondary atom site location: difference Fourier map
*R*[*F*^2^ > 2σ(*F*^2^)] = 0.083	Hydrogen site location: geom and difmap
*wR*(*F*^2^) = 0.189	H-atom parameters constrained
*S* = 1.27	*w* = 1/[σ^2^(*F*_o_^2^) + (0.0626*P*)^2^ + 1.2781*P*] where *P* = (*F*_o_^2^ + 2*F*_c_^2^)/3
3271 reflections	(Δ/σ)_max_ = 0.001
237 parameters	Δρ_max_ = 0.24 e Å^−^^3^
0 restraints	Δρ_min_ = −0.29 e Å^−^^3^

### Special details

**Table:**

Geometry. All e.s.d.'s (except the e.s.d. in the dihedral angle between two l.s. planes) are estimated using the full covariance matrix. The cell e.s.d.'s are taken into account individually in the estimation of e.s.d.'s in distances, angles and torsion angles; correlations between e.s.d.'s in cell parameters are only used when they are defined by crystal symmetry. An approximate (isotropic) treatment of cell e.s.d.'s is used for estimating e.s.d.'s involving l.s. planes.
Refinement. Refinement of *F*^2^ against ALL reflections. The weighted *R*-factor *wR* and goodness of fit *S* are based on *F*^2^, conventional *R*-factors *R* are based on *F*, with *F* set to zero for negative *F*^2^. The threshold expression of *F*^2^ > σ(*F*^2^) is used only for calculating *R*-factors(gt) *etc*. and is not relevant to the choice of reflections for refinement. *R*-factors based on *F*^2^ are statistically about twice as large as those based on *F*, and *R*- factors based on ALL data will be even larger.

### Fractional atomic coordinates and isotropic or equivalent isotropic displacement parameters (Å^2^)

**Table:**

	*x*	*y*	*z*	*U*_iso_*/*U*_eq_	
O1	0.9740 (2)	0.97287 (14)	0.3245 (2)	0.0657 (7)	
H1	1.0093	0.9797	0.2760	0.098*	
O2	0.9063 (2)	0.51536 (14)	0.3293 (2)	0.0608 (6)	
O3	0.82019 (18)	0.56944 (13)	0.45749 (18)	0.0488 (5)	
C1	0.9519 (3)	0.87795 (19)	0.3346 (3)	0.0447 (7)	
C2	0.8980 (3)	0.8521 (2)	0.4176 (3)	0.0545 (8)	
H2	0.8777	0.8996	0.4631	0.065*	
C3	0.8744 (3)	0.75689 (19)	0.4330 (3)	0.0461 (7)	
H3	0.8381	0.7403	0.4890	0.055*	
C4	0.9044 (2)	0.68486 (19)	0.3652 (2)	0.0370 (6)	
C5	0.9577 (3)	0.7118 (2)	0.2820 (3)	0.0460 (7)	
H5	0.9781	0.6646	0.2362	0.055*	
C6	0.9810 (3)	0.8072 (2)	0.2660 (3)	0.0467 (7)	
H6	1.0162	0.8242	0.2093	0.056*	
C7	0.8785 (2)	0.5822 (2)	0.3806 (3)	0.0403 (7)	
C8	0.7870 (3)	0.4715 (2)	0.4759 (3)	0.0527 (8)	
H8A	0.8560	0.4335	0.5156	0.063*	
H8B	0.7463	0.4414	0.3997	0.063*	
C9	0.7087 (4)	0.4766 (2)	0.5519 (3)	0.0672 (10)	
H9A	0.7509	0.5067	0.6277	0.081*	
H9B	0.6418	0.5170	0.5124	0.081*	
C10	0.6668 (4)	0.3798 (3)	0.5749 (4)	0.0862 (13)	
H10A	0.6203	0.3516	0.5006	0.129*	
H10B	0.6202	0.3864	0.6270	0.129*	
H10C	0.7327	0.3389	0.6120	0.129*	
O4	0.5717 (2)	0.61479 (15)	0.7201 (2)	0.0742 (8)	
H4	0.5298	0.6082	0.7622	0.111*	
O5	0.5895 (2)	1.07375 (15)	0.6687 (2)	0.0594 (6)	
O6	0.68661 (18)	1.01843 (13)	0.55031 (18)	0.0492 (5)	
C11	0.5838 (3)	0.7104 (2)	0.7005 (3)	0.0482 (7)	
C12	0.6369 (3)	0.7352 (2)	0.6165 (3)	0.0610 (9)	
H12	0.6633	0.6871	0.5766	0.073*	
C13	0.6507 (3)	0.8303 (2)	0.5919 (3)	0.0497 (8)	
H13	0.6860	0.8462	0.5349	0.060*	
C14	0.6125 (2)	0.9033 (2)	0.6512 (2)	0.0385 (6)	
C15	0.5595 (3)	0.8775 (2)	0.7353 (3)	0.0441 (7)	
H15	0.5335	0.9256	0.7757	0.053*	
C16	0.5447 (3)	0.7818 (2)	0.7602 (3)	0.0470 (7)	
H16	0.5088	0.7656	0.8166	0.056*	
C17	0.6265 (2)	1.0062 (2)	0.6259 (2)	0.0403 (7)	
C18	0.7092 (3)	1.11661 (19)	0.5211 (3)	0.0476 (7)	
H18A	0.7597	1.1491	0.5916	0.057*	
H18B	0.6365	1.1524	0.4918	0.057*	
C19	0.7673 (3)	1.1111 (2)	0.4270 (3)	0.0526 (8)	
H19A	0.7162	1.0776	0.3577	0.063*	
H19B	0.8390	1.0741	0.4571	0.063*	
C20	0.7954 (3)	1.2110 (2)	0.3895 (3)	0.0672 (10)	
H20A	0.7243	1.2470	0.3571	0.101*	
H20B	0.8337	1.2047	0.3300	0.101*	
H20C	0.8461	1.2443	0.4579	0.101*	

### Atomic displacement parameters (Å^2^)

**Table:**

	*U*^11^	*U*^22^	*U*^33^	*U*^12^	*U*^13^	*U*^23^
O1	0.1019 (19)	0.0285 (11)	0.0922 (17)	−0.0029 (11)	0.0670 (15)	0.0030 (11)
O2	0.0824 (16)	0.0342 (11)	0.0856 (16)	−0.0030 (10)	0.0544 (14)	−0.0113 (11)
O3	0.0671 (14)	0.0298 (10)	0.0629 (13)	−0.0052 (9)	0.0393 (11)	−0.0006 (9)
C1	0.0551 (18)	0.0306 (14)	0.0554 (18)	−0.0010 (12)	0.0275 (15)	0.0017 (12)
C2	0.078 (2)	0.0349 (15)	0.069 (2)	0.0015 (15)	0.0485 (18)	−0.0054 (14)
C3	0.0614 (19)	0.0340 (15)	0.0558 (18)	−0.0001 (13)	0.0370 (16)	0.0031 (12)
C4	0.0394 (15)	0.0325 (14)	0.0419 (15)	−0.0006 (11)	0.0168 (12)	−0.0009 (11)
C5	0.0575 (18)	0.0392 (15)	0.0520 (17)	−0.0024 (13)	0.0325 (15)	−0.0083 (13)
C6	0.0606 (19)	0.0425 (16)	0.0486 (17)	−0.0022 (14)	0.0337 (15)	0.0032 (13)
C7	0.0398 (15)	0.0372 (15)	0.0479 (16)	0.0008 (12)	0.0198 (13)	−0.0033 (12)
C8	0.068 (2)	0.0302 (15)	0.066 (2)	−0.0044 (14)	0.0305 (17)	0.0049 (13)
C9	0.101 (3)	0.0448 (18)	0.072 (2)	−0.0123 (18)	0.051 (2)	−0.0045 (16)
C10	0.121 (4)	0.063 (2)	0.099 (3)	−0.018 (2)	0.070 (3)	0.000 (2)
O4	0.107 (2)	0.0348 (12)	0.111 (2)	−0.0041 (12)	0.0776 (17)	0.0060 (12)
O5	0.0821 (16)	0.0367 (12)	0.0756 (15)	0.0051 (10)	0.0478 (13)	−0.0039 (10)
O6	0.0660 (13)	0.0292 (10)	0.0655 (13)	−0.0020 (9)	0.0395 (11)	−0.0008 (9)
C11	0.0502 (17)	0.0375 (15)	0.0637 (19)	−0.0021 (13)	0.0279 (15)	0.0039 (14)
C12	0.079 (2)	0.0347 (16)	0.093 (3)	0.0007 (15)	0.061 (2)	−0.0071 (16)
C13	0.0618 (19)	0.0394 (16)	0.065 (2)	−0.0035 (14)	0.0435 (17)	−0.0034 (14)
C14	0.0347 (14)	0.0397 (15)	0.0427 (15)	−0.0020 (11)	0.0145 (12)	−0.0007 (12)
C15	0.0544 (18)	0.0368 (15)	0.0475 (16)	0.0010 (13)	0.0254 (14)	−0.0052 (12)
C16	0.0520 (17)	0.0494 (17)	0.0483 (17)	0.0005 (14)	0.0281 (15)	0.0023 (14)
C17	0.0412 (15)	0.0390 (15)	0.0422 (15)	0.0020 (12)	0.0154 (13)	−0.0028 (12)
C18	0.0585 (19)	0.0271 (14)	0.0614 (19)	−0.0043 (12)	0.0250 (15)	−0.0014 (13)
C19	0.063 (2)	0.0368 (16)	0.067 (2)	−0.0039 (14)	0.0330 (17)	−0.0001 (14)
C20	0.081 (3)	0.0498 (19)	0.084 (3)	−0.0122 (18)	0.045 (2)	0.0050 (18)

### Geometric parameters (Å, °)

**Table:**

O1—C1	1.353 (3)	O4—C11	1.360 (3)
O1—H1	0.8206	O4—H4	0.8191
O2—C7	1.210 (3)	O5—C17	1.213 (3)
O3—C7	1.326 (3)	O6—C17	1.328 (3)
O3—C8	1.449 (3)	O6—C18	1.449 (3)
C1—C2	1.384 (4)	C11—C16	1.381 (4)
C1—C6	1.385 (4)	C11—C12	1.383 (4)
C2—C3	1.374 (4)	C12—C13	1.369 (4)
C2—H2	0.9300	C12—H12	0.9300
C3—C4	1.397 (4)	C13—C14	1.389 (4)
C3—H3	0.9300	C13—H13	0.9300
C4—C5	1.386 (4)	C14—C15	1.387 (4)
C4—C7	1.478 (4)	C14—C17	1.476 (4)
C5—C6	1.375 (4)	C15—C16	1.381 (4)
C5—H5	0.9300	C15—H15	0.9300
C6—H6	0.9300	C16—H16	0.9300
C8—C9	1.497 (4)	C18—C19	1.492 (4)
C8—H8A	0.9700	C18—H18A	0.9700
C8—H8B	0.9700	C18—H18B	0.9700
C9—C10	1.488 (5)	C19—C20	1.523 (4)
C9—H9A	0.9700	C19—H19A	0.9700
C9—H9B	0.9700	C19—H19B	0.9700
C10—H10A	0.9600	C20—H20A	0.9600
C10—H10B	0.9600	C20—H20B	0.9600
C10—H10C	0.9600	C20—H20C	0.9600
			
C1—O1—H1	109.5	C11—O4—H4	109.5
C7—O3—C8	117.4 (2)	C17—O6—C18	117.6 (2)
O1—C1—C2	117.5 (3)	O4—C11—C16	122.4 (3)
O1—C1—C6	122.8 (3)	O4—C11—C12	117.6 (3)
C2—C1—C6	119.7 (3)	C16—C11—C12	120.0 (3)
C3—C2—C1	120.4 (3)	C13—C12—C11	120.4 (3)
C3—C2—H2	119.8	C13—C12—H12	119.8
C1—C2—H2	119.8	C11—C12—H12	119.8
C2—C3—C4	120.4 (3)	C12—C13—C14	120.7 (3)
C2—C3—H3	119.8	C12—C13—H13	119.7
C4—C3—H3	119.8	C14—C13—H13	119.7
C5—C4—C3	118.5 (2)	C15—C14—C13	118.4 (3)
C5—C4—C7	120.6 (2)	C15—C14—C17	120.1 (2)
C3—C4—C7	120.8 (2)	C13—C14—C17	121.5 (2)
C6—C5—C4	121.2 (3)	C16—C15—C14	121.3 (3)
C6—C5—H5	119.4	C16—C15—H15	119.3
C4—C5—H5	119.4	C14—C15—H15	119.3
C5—C6—C1	119.8 (3)	C15—C16—C11	119.3 (3)
C5—C6—H6	120.1	C15—C16—H16	120.4
C1—C6—H6	120.1	C11—C16—H16	120.4
O2—C7—O3	122.2 (3)	O5—C17—O6	122.3 (3)
O2—C7—C4	124.8 (3)	O5—C17—C14	125.3 (3)
O3—C7—C4	113.0 (2)	O6—C17—C14	112.4 (2)
O3—C8—C9	107.6 (2)	O6—C18—C19	107.3 (2)
O3—C8—H8A	110.2	O6—C18—H18A	110.3
C9—C8—H8A	110.2	C19—C18—H18A	110.3
O3—C8—H8B	110.2	O6—C18—H18B	110.3
C9—C8—H8B	110.2	C19—C18—H18B	110.3
H8A—C8—H8B	108.5	H18A—C18—H18B	108.5
C10—C9—C8	112.5 (3)	C18—C19—C20	111.8 (3)
C10—C9—H9A	109.1	C18—C19—H19A	109.3
C8—C9—H9A	109.1	C20—C19—H19A	109.3
C10—C9—H9B	109.1	C18—C19—H19B	109.3
C8—C9—H9B	109.1	C20—C19—H19B	109.3
H9A—C9—H9B	107.8	H19A—C19—H19B	107.9
C9—C10—H10A	109.5	C19—C20—H20A	109.5
C9—C10—H10B	109.5	C19—C20—H20B	109.5
H10A—C10—H10B	109.5	H20A—C20—H20B	109.5
C9—C10—H10C	109.5	C19—C20—H20C	109.5
H10A—C10—H10C	109.5	H20A—C20—H20C	109.5
H10B—C10—H10C	109.5	H20B—C20—H20C	109.5

### Hydrogen-bond geometry (Å, °)

**Table:**

*D*—H···*A*	*D*—H	H···*A*	*D*···*A*	*D*—H···*A*
O4—H4···O5^i^	0.82	1.93	2.730 (3)	167
O1—H1···O2^ii^	0.82	1.91	2.720 (3)	171

Symmetry codes: (i) −*x*+1, *y*−1/2, −*z*+3/2; (ii) −*x*+2, *y*+1/2, −*z*+1/2.
